# Effects of a 6-Week Faroese Chain Dance Programme on Postural Balance, Physical Function, and Health Profile in Elderly Subjects: A Pilot Study

**DOI:** 10.1155/2019/5392970

**Published:** 2019-07-17

**Authors:** Jóhan Hofgaard, Georgios Ermidis, Magni Mohr

**Affiliations:** ^1^Centre of Health Science, Faculty of Health Sciences, University of the Faroe Islands, Tórshavn, Faroe Islands; ^2^Department of Movement and Wellness Sciences, University of Parthenope, Naples, Italy; ^3^Department of Sports Science and Clinical Biomechanics, SDU Sport and Health Sciences Cluster (SHSC), Faculty of Health Sciences, University of Southern Denmark, Odense, Denmark; ^4^Center for Health and Performance, Department of Food and Nutrition, and Sport Science, University of Gothenburg, Gothenburg, Sweden

## Abstract

The present pilot study investigates the impact of a Faroese chain dance intervention on health profile, mobility, and postural balance in elderly subjects. Healthy elderly subjects (n=27; age 75 ± 5 yrs) were randomised into an intervention group (IG) and a control group (CG). IG performed twice-weekly sessions of Faroese chain dance over 6 weeks. Dancing sessions lasted 30 min in the initial 3 weeks and 45 min in the final 3 weeks. Health profile was determined before and after intervention by measuring blood pressure, resting heart rate, muscle mass, and body fat content. Postural balance was evaluated using the Berg Balance Scale (BBS) and Fullerton Advanced Balance Scale (FAB) tests, while mobility was assessed using the Short Physical Performance Battery (SPPB), the Timed Up & Go (TUG) test, the 6-min walk test, and the 30-s sit-to-stand test. Systolic and diastolic blood pressure were lowered (9 ± 6 and 6 ± 3 mmHg, respectively) in IG, with a tendency (P=0.07) for a greater change score than in CG. Mean arterial pressure declined (P<0.05) by 7 ± 3 mmHg in IG, which tended (P=0.09) to be greater than in CG. IG improved (P<0.05) on BBS and FAB scores by 3.6 ± 2.1% and 15.8 ± 8.3%, with the change score for FAB being greater (P<0.05) than in CG (0.3 ± 1.6). Moreover, the postintervention SPPB score was improved (P<0.05) more in IG (13.9 ± 7.4%) compared to CG, while performance in the 30-s sit-to-stand, 6-min walk, and TUG tests improved (4–15%; P<0.05) in IG only. Body fat content was reduced (P<0.05) from 36.3 ± 2.8% to 34.8 ± 2.8% in IG, with no between-group differences and no change in CG (34.1 ± 2.8% to 33.7 ± 3.1%). In conclusion, a 6-week Faroese chain dance programme lowers blood pressure and improves postural balance and physical function in elderly.

## 1. Introduction

Prolonged physical inactivity leads to a deterioration in health status, including cardiovascular disease [1], obesity [2], metabolic deficiencies [3], and frailty [4]. Physical activity has a health-promoting effect, such as improvements in cardiovascular function [5], postural balance and physical capacity [6], mental health [7, 8], and body composition [9]. The impaired health conditions provoked by physical inactivity and the beneficial impact of physical activity suggest that further efforts are required to encourage physical activity in inactive and frail populations such as the elderly [10].

Physical inactivity is commonly observed in the elderly, with, for example, 27% of men and women aged 65–74 and 46% of men and 65% of women older than 75 years of age being physically inactive in England [11–14]. The barriers to physical activity for elderly people have been shown to be chronic conditions, bad weather, programme costs, reliability of affordable transport, low self-efficacy, and lack of interest and motivation [15, 16]. Enjoyment and motivation to maintain a good health status have been demonstrated to be essential reasons for promoting physical activity in the elderly population [16].

Physical inactivity defined as being physically active at moderate exercise intensity for less than 2.5 hours a week [17]. It is a major burden on the health sector. If we consider a relatively small country such as Denmark, the cost to society of treating and caring for physically inactive elderly people was estimated to be around EUR 500,000 million in 2013 alone [18]. Moreover, in Denmark the percentage of the population aged over 65 years has increased by a third in the last decade, so it may be presumed that physically inactive elderly people cost society even more today due to the major demographic changes [19]. If the elderly population were more engaged in physical activities, it would be highly beneficial for the human and society factor.

Elderly individuals display an interest, especially if they have previous experience, in various challenging activities, including dancing [20]. Dancing improves social interaction and enjoyment (Guzmán-García et al., 2012) and may break down barriers to physical activity in the elderly population. Pau et al. [21] demonstrated that vigorous physical activity has a positive effect on static and dynamic motor tasks in elderly, while light activity showed acceptable results for static balance. Finally, findings suggest that dancing, regardless of the style can induce broad-spectrum effects, such as improving muscular strength, postural balance, endurance, and general fitness in the elderly [22].

In the Faroe Islands, a small-scale island society in the North Atlantic Ocean, a simple ancient chain dance, dating back to medieval times, where the dancers sing ancient Nordic ballads while dancing sideways, is an integral part of Faroese culture that persists today. Ballads are taught in primary school every year in the weeks prior to the Christian period of Shrovetide. The dance is normally performed at celebratory events, such as weddings, festivals, and national events, and is carried out sporadically when large crowds are gathered [23]. However, the number of events and the number of participants have been declining over the last few decades. Some Faroese chain dance clubs are active around the islands, and it is mainly elderly people who attend weekly dances and events.

The physical, mental, and social effects of the Faroese chain dance have not yet been examined scientifically. The purpose of the present study is therefore to test the hypothesis that a 6-week Faroese chain dance programme will improve general health status, postural balance, and physical function in elderly subjects.

## 2. Methods

### 2.1. Participants

Fifty-nine participants (36 women and 23 men) originally volunteered to participate in the study. The inclusion criteria were aged over 67; able to dance for 30 min; and absence of dementia. Given that nearly half of the elderly population has two or more chronic conditions, it is recommended that adults with chronic conditions should not be excluded [22]. However, participants were excluded if they took part in organized physical activity once a week or more. Two volunteers from the original sample were younger than 67, eight could not dance for 30 min, and 16 suffered from dementia, as determined by the staff at their nursing homes. These participants were excluded from the study. The study applied a randomised controlled trial design with a dance-based intervention. Thus, the 33 participants who met the inclusion criteria were randomly and gender-specifically assigned to an intervention group (IG) or a control group (CG) stratified for age. Six participants (2 from IG, 4 from CG) dropped out prior to the pretests due to time constraints and travel expenses. One participant from CG got sick and did not complete the posttest, and one from CG participated in the dancing three times without the test supervisor realising and was subsequently excluded from the study sample. The final sample was therefore 15 participants in IG (6 males; 9 females) and 10 in CG (3 males; 7 females), which based on power calculations of primary outcomes such as blood pressure, muscle mass, and performance tests gives sufficient statistical power (0.8) to demonstrate potential within and between-group differences (P<0.05). The characteristics of the participants are displayed in Table 1. All the participants were thoroughly informed of potential risks and discomforts associated with the experiment before giving their written consent to participate according to the guidelines of the Helsinki Declaration. The study was approved by the local ethics committee of the Faroe Islands.

### 2.2. Experimental Procedure

Baseline data were collected in the week prior to a Faroese chain dance intervention and the posttests were conducted in the week after the intervention. For the physical tests, the procedures were explained and demonstrated prior to each test. In addition, a familiarisation test trial was organized prior to baseline testing. Pre- and posttests were performed at the same time of day.

### 2.3. Experimental Protocol

The first assessments comprised blood pressure and resting heart rate measurements. The cuff of a digital blood pressure monitor (AND, UA-779, Abingdon, United Kingdom) was strapped around the left upper arm 2–3 cm above cubital fossa. The participants were instructed to sit upright without crossing their legs for 5 min. Three measurements were conducted with 1 min. of rest in between, and the average was used as the test result. Height, weight, body fat percentage, and muscle mass were measured using a Body Composition Analyzer (InBody 270, Seoul, South Korea) [24]. The participants were instructed to remove their socks and step on the scale with their feet aligned with the foot electrodes while holding the handle with thumbs placed on the oval electrodes and arms straight out from the body. Four participants (2 IG, 2 CG) had pacemakers and could not be measured because the device sends electrical current through the body, which can tamper with a pacemaker.

The participants performed two balance tests and were scored for each test. The first test, the Berg Balance Scale (BBS) test, was developed to measure balance in older people with impairment in balance function. This test is currently the most commonly used clinical test for assessing balance ability in the elderly. The second test, the Fullerton Advanced Balance Scale (FAB) test, identifies balance deficits in the elderly. The BBS and FAB tests have both been shown to be able to predict fall risk in the elderly [25, 26]. However, the BBS test has been shown to be less predictive because of a ceiling effect [27]. The FAB test was developed as a performance-based measure to assess the subtle changes in multiple dimensions of balance ability [28]. The FAB scale is recommended for predicting the fall risk in higher-functioning elderly people, as it includes criteria for evaluating multiple dimensions of balance ability [25].

To evaluate mobility and dynamic balance, the participants performed the Timed Up & Go (TUG) test, where they were seated and on the word “go” stood up from the chair, walked 3 m, turned around, walked back again, and sat down. They were timed from the word go until seated again. The TUG test has proven sensitive for detecting change in performance [29].

Moreover, to evaluate mobility in the lower extremities, the participants performed the Short Physical Performance Battery (SPPB) [30]. The reliability of the SPPB for frail participants has been shown to be acceptable, and it can be used to monitor meaningful changes in functional capacity in vulnerable groups [30].

To evaluate strength in the lower extremities, the participants performed a 30-s sit-to-stand test, where they had 30 s to complete as many stands from a fully seated position as possible. The five-repetition sit-to-stand test is a widely used measure of functional strength, particularly in the elderly. The test-retest reliability of the test has been classified as good-to-high in most populations and settings [31].

To evaluate endurance, the participants performed a 6-min walk test, where they walked around a room following markers on the floor as fast and for as long as they could. The total distance covered was registered as the test result. The 6-min walk test (6 MWT) is an inexpensive, reliable, and valid test of mobility and submaximal work capacity in seniors [32].

IG participated in a six-week Faroese chain dance intervention period. Pre- and posttesting were performed. The participants met at an indoor recreation centre twice a week to dance on a wooden surface. Faroese chain dance is a medieval ring dance. It is organized as a chain where all dancers are positioned side-by-side holding hands firmly with the dancer to the left and right forming a ring or circle. All dancers face the centre of the ring while dancing. The hands are held tightly with a 90° flexion in the elbow joint. These static muscle contractions in the arms and shoulders give support to maintain an upright posture and provides balance to the dancers. The dancers then move rhythmically and simultaneously sideways taking two relatively forceful steps to the left and one to the right with the entire chain is moving together. The dancers sing ballads and dance to the pace or tempo of the songs, which can be highly variable and is affected by factors such as the type of ballad and the geographical region of the dancers/singers. Normally, the metronome number of quarter notes or dotted quarter notes of the ballads used in Faroese chain dancing is approximately 80-105 bts·min^−1^ (personal communication). Mainly, the ballads concern Nordic historical or mythological events often involving heroism. The tempo in the present dance intervention was controlled by 2–5 experienced external dancers, who chose the ballads and lead the dancing and singing. The tempo was estimated to be ~90 bts·min^−1^. In the first 3 weeks, the participants danced continuously for 30 min and in the last 3 weeks for 45 min. CG were encouraged to continue their normal daily routines during the intervention period.

### 2.4. Statistical Analysis

The normality of the distribution of outcome measures was tested using the Shapiro-Wilk test and Q-Q plots. If the results were nonnormally distributed, a Mann-Whitney U-test was performed to examine possible between-group differences at baseline. Independent samples t-tests were used for the normally distributed results to examine possible between-group differences in baseline scores prior to the intervention. Two-way repeated measures ANOVAs were performed to test the main effects of time (pre; post) and group (CG; IG) and time-by-group interactions. When a significant interaction was observed, a Tukey post hoc test with Bonferroni correction was used to identify the points of difference. Significance was accepted at* P* < 0.05. Data are reported as means +/-SD. The data were analyzed using Statistical Package of Social Sciences (SPSS) version 25.

## 3. Results

### 3.1. Blood Pressure and Resting Heart Rate

**Figure 1:**
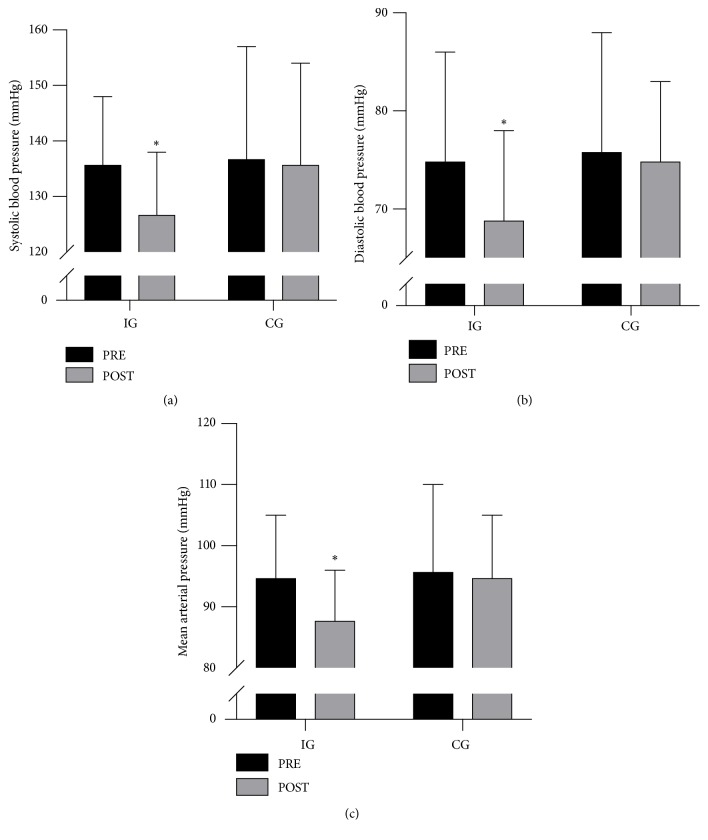
Systolic (a), diastolic (b), and mean arterial pressure (c) at baseline and after the intervention period in the intervention group (IG; n=15) and the control group (CG; n=10). *∗* denotes a significant difference from baseline. Significance level; P<0.05.

Systolic blood pressure (SBP) and diastolic blood pressure (DBP) were reduced (P<0.05) in IG by 9 ± 6 and 6 ± 3 mmHg, respectively, which was not different from the change score in CG (-1 ± 8 and 0 ± 4 mmHg). However, the change score for DBP in IG tended (P=0.07) to be greater than in CG (Figure 1). Mean arterial pressure (MAP) was lowered (P<0.05) by 7 ± 3 mmHg in IG, which tended (P=0.09) to be greater than in CG (Figure 1). Resting heart rate (RHR) was 66 ± 6 and 70 ± 4 bpm at baseline in IG and CG, respectively, and did not change during the intervention period.

### 3.2. Postural Balance and Physical Performance

**Figure 2:**
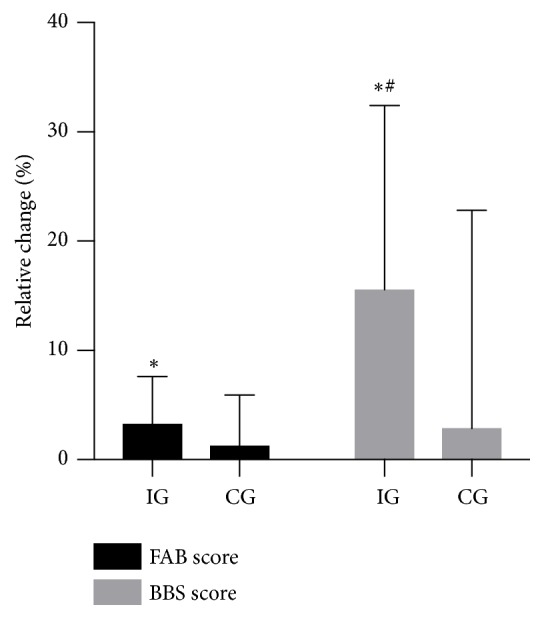
Relative change in the Berg Balance Scale (BBS) and Fullerton Advanced Balance Scale (FAB) during the intervention period in the intervention group (IG; n=15) and the control group (CG; n=10). *∗* denotes a significant difference from baseline. # denotes a significant difference in change score from CG. Significance level; P<0.05.

**Figure 3:**
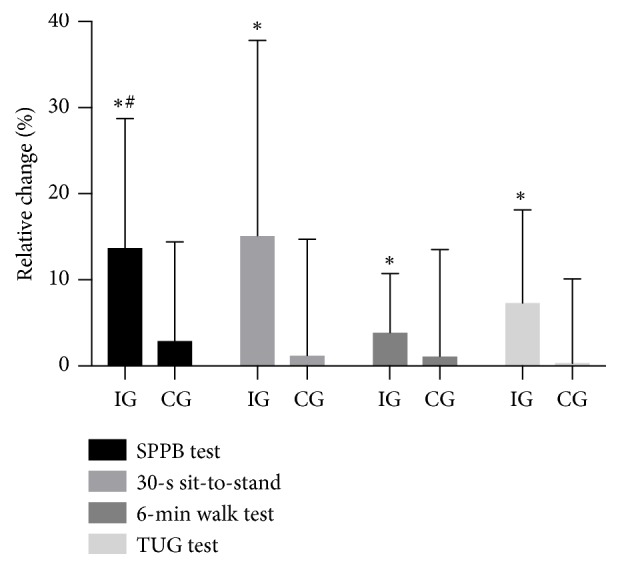
Relative change in the Short Physical Performance Battery (SPPB), the Timed Up & Go (TUG) test, the 6-min walk test and the 30-s sit-to-stand test during the intervention period in the intervention group (IG; n=15), and the control group (CG; n=10). *∗* denotes a significant difference from baseline. # denotes a significant difference in change score from CG. Significance level; P<0.05.

IG improved (P<0.05) their BBS score by 1.8 ± 1.0, corresponding to 3.6 ± 2.1%, during the intervention period, with no difference from CG (0.7 ± 1.1; Figure 2). Moreover, the FAB score was increased (P<0.05) by 3.7 ± 1.7 (15.8 ± 8.3%) in IG after intervention, with a greater change score (P<0.05) than in CG (0.3 ± 1.6) (Figure 2). The SPPB score was improved (P<0.05) more in IG (1.3 ± 0.6 or 13.9 ± 7.4%) after intervention than in CG (0.1 ± 0.4; Figure 3). In the 30-s sit-to-stand test, IG had improved (P<0.05) by 2.3 ± 1.7 repetitions (15.3 ± 11.3%) after intervention, with no between-group difference or change in CG (0.1 ± 0.3; Figure 3). 6-min walk test performance was improved (P<0.05) by 17 ± 14 m (4.1 ± 3.3%), with no between-group difference and no change in CG (2 ± 22 m; Figure 3). TUG performance was increased (P<0.05) by 0.58 ± 0.39 s (7.5 ± 5.3%) in IG after intervention, with no between-group difference and no change in CG (0.04 ± 0.30 s; Figure 3).

### 3.3. Body Composition

**Figure 4:**
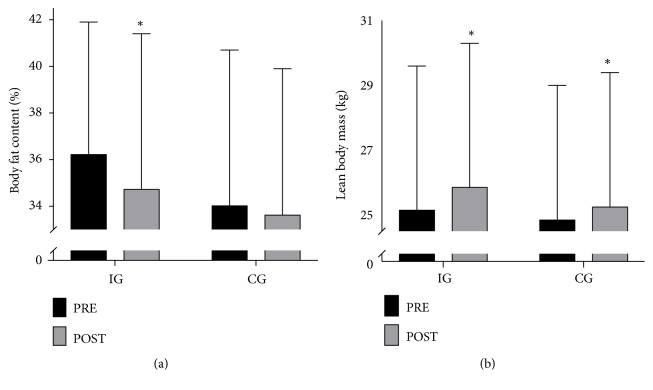
Body fat content (a) and lean body mass (b) at baseline and after the intervention period in the intervention group (IG; n=15) and the control group (CG; n=10). *∗* denotes a significant difference from baseline. Significance level; P<0.05.

Body mass was unchanged in both IG (74.6 ± 6.4 vs. 74.8 ± 6.6 kg) and CG (72.3 ± 6.1 vs. 72.4 ± 5.8 kg) over the intervention period. However, total body fat content was reduced (P<0.05) from 36.3 ± 2.8% to 34.8 ± 2.8% in IG, with no between-group differences and no change in CG (34.1 ± 2.8 to 33.7 ± 3.1%; Figure 4(a)). LBM was elevated (P<0.05) by 0.7 ± 0.5 and 0.4 ± 0.5 kg in IG and CG after intervention, with no between-group difference (Figure 4(b)).

## 4. Discussion

The present study is the first to investigate the impact of a 6-week Faroese chain dance intervention on health status, postural balance, and physical function in elderly subjects. The major findings were that the dance intervention improved postural balance and physical function, as well as augmented health parameters.

We hypothesised that a structured and supervised Faroese chain dance programme would improve postural balance in the healthy elderly. Thus, the hypothesis was verified, with a 4 and 16% increase in BBS and FAB test performance as markers of postural balance and resistance to fall risk [25]. These findings are consistent with other studies. Indeed, one study demonstrated that folklore dancing organized as 1-hour sessions three times a week over 8 weeks had a beneficial effect on BBS test performance [33]. Moreover, other types of dance, such as jazz dancing, over a period of 15 weeks (90-min weekly sessions) improved static balance [34]. In addition, a variety of ballroom dances conducted for 1 hour twice weekly over 12 weeks upregulated postural balance [35]. As far as we know, the FAB test has not been applied previously in dance intervention studies. However, Pirouzi et al. [36] also demonstrated improvements in both FAB and BBS scores in elderly subjects who performed 30 min of treadmill walking (three times weekly in 4 weeks). This study is comparable to our study in terms of training volume and frequency in relation to the dose response. However, Faroese chain dancing differs markedly from straight line walking. The dancing steps are performed relatively forcefully sideways, a movement not normally performed, and with markedly greater effort than normal steps. Moreover, there is a constant static involvement of the upper-body due the tight “hand-holding”, which enable the dancers, even frail groups, to maintain a straight upright posture that is likely to continuously activate core muscles. Thus, the unorthodox locomotion pattern may be beneficial in terms of improving postural balance. In comparison, several studies on elderly participants using recreational football training and walking football, which also has an unorthodox locomotion pattern, as intervention method, have demonstrated that components of postural balance are markedly improved after periods lasting 12-16 weeks [37, 38]

The statistical analysis of time-by-group showed a significant difference in SPPB performance and in effect of time a significant difference for SPPB, TUG, the 6-min walk test, and the 30-s sit-to-stand test. Thus, different types of physical performance were upregulated after the chain dance intervention by 4–15%. The present study is the first dance intervention study to our knowledge to apply the SPPB test. However, it has been demonstrated that both resistance and aerobic training in the elderly increase SPPB performance over a 5-month period of 3–4 weekly sessions [39]. Moreover, a culturally specific dance intervention improved functional capacity in African-American women aged 36–82 yrs [40]. Also, Tai Chi performed by elderly subjects for 1 hour twice a week over 12 weeks has been shown to induce an improvement in SPPB score [41]. In the TUG test, the intervention group improved performance by 8%, despite there being no difference from the control group. This finding is confirmed by an RCT study by Hui et al. [42] showing a marked improvement in the TUG test over a 12-week dance intervention. They applied two 50-min weekly sessions for the first 6 weeks followed by two 60-min sessions per week for the last 6 weeks. Thus, if the Faroese chain dance training was extended for additional 6 weeks, it might show an effect on time-by-group for TUG performance. The same pattern was seen for the 6-min walk test and the 30-s sit-to-stand test, where clear within-group improvements were observed but no between-group differences. Due to the exclusion criteria and drop-outs, the final sample size was relatively low, which may have caused a statistical type II error in relation to the between-group comparison. However, collectively a 6-week intervention involving Faroese chain dance improves physical function in elderly subjects.

Systolic and diastolic blood pressure were lowered by 9 and 6 mmHg, respectively, after 6 weeks of Faroese chain dance. The change score did not differ from the control group, but a strong tendency was observed (P<0.07 and 0.09) for the change in diastolic blood pressure and mean arterial pressure (-7 mmHg), respectively. The magnitude of reduction in arterial blood pressure is comparable with the acute effect of taking one standard dose of a blood pressure-lowering drug and is of major clinical importance because a blood pressure reduction of such a magnitude corresponds to a 20%–30% lower risk of stroke in hypertensive individuals [43]. A few other studies have applied dancing as an exercise training protocol. For example, dancing with an aerobic component was shown in a RCT to cause positive adaptations in women older than 65 years with arterial hypertension [44]. In addition, Zumba dancing for hospital employees has been demonstrated to decrease the degree of arterial hypertension markedly [37]. However, the literature is not consistent. In a study by Kim et al. [45], the participants completed one hour of cha-cha dancing twice weekly for 6 months and showed no differences in systolic and diastolic blood pressure. This finding may relate to the low intensity of the dance exercise protocol in relation to the fitness status of the group of participants. Arterial hypertension is stated to be a principal risk factor for cardiovascular and renal diseases. Given the increasing prevalence of hypertension with age and an increasingly aging population [46], treatment of elderly patients with arterial hypertension will become increasingly important in the future. Faroese chain dance may be an alternative training protocol for elderly or frail patients with arterial hypertension.

Body fat content was reduced in IG only, in line with several other studies using dance as an exercise training intervention [37, 41, 44], but in disagreement with others [45, 47]. In contrast, lean body mass was elevated after intervention in both IG (0.7 kg) and CG (0.4 kg). This may relate to the use of a bioelectrical impedance analysis device [48] rather than the gold standard DXA scans. The InBody technology that was applied in the present study has a bias of 2.5% and a 95% limit of agreement from -10.02 to 4.96 (Montgomery et al., 2017), which may be a limitation in the present study.

Faroese chain dance has a beneficial effect on postural balance and physical function, and most likely on blood pressure and body fat content, in elderly participants after only 6 weeks of training. The chain dance is a type of physical activity in which anyone capable of walking can take part. In addition, the dancers hold hands during dancing, with one partner on each side, which provides support and minimises the risk of falls. Thus, it can be considered a safe physical activity for frail groups such as the elderly and can for example be organized in nursing homes. Finally, the dance and the corresponding ballads are an integral part of Faroese identity and culture and have a strong social component among the population, which makes it an exercise training method well suited for the elderly population for this ethnic group. Therefore, it can be recommended that the growing elderly population might participate in this form of physical activity on a regular basis in order to improve or maintain postural balance and physical function, as well as general health status. Future studies should aim to test the benefits of this type of physical activity in other frail participant groups, such as patients in hospitals, and to describe the physiological response to a Faroese chain dance session. A limitation in the present study is the relatively low sample size despite the fact that fifteen and ten participants in the intervention and control group, respectively, provide an acceptable statistical power for the primary outcomes of the study. Thus, it is recommended to evaluate the current intervention in a large sample size study in the future.

## 5. Conclusion

In conclusion, six weeks of Faroese chain dance training induced improvements in postural balance and physical function, as well as general health status, in elderly subjects.

## Figures and Tables

**Table 1:** Participant characteristics.

	Age (yrs)	Height (cm)	Weight (kg)	Body fat (%)	MAP (mmHg)
IG (n=15)	75 ± 5	165 ± 9	74.6 ± 12.8	36.5 ± 5.6	95 ± 10
CG (n=10)	74 ± 4	165 ± 9	72.3 ± 12.3	34.1 ± 6.6	96 ± 14

Age, height, weight, body fat, and mean arterial pressure (MAP) in intervention group (IG; n=15) and control group (CG; n=10) at baseline. Data are means ± SD.

## Data Availability

The data used to support the findings of this study are available from the corresponding author upon request.
